# The increased risk of active tuberculosis disease in patients with dermatomyositis – a nationwide retrospective cohort study

**DOI:** 10.1038/srep16303

**Published:** 2015-11-17

**Authors:** Ping-Hsun Wu, Yi-Ting Lin, Yi-Hsin Yang, Yu-Chih Lin, Yi-Ching Lin

**Affiliations:** 1 Division of Nephrology, Department of Internal Medicine, Kaohsiung Medical University Hospital, Kaohsiung, Taiwan; 2 Faculty of Internal Medicine, College of Medicine, Kaohsiung Medical University, Kaohsiung, Taiwan; 3 Department of Family Medicine, Kaohsiung Municipal Hsiao–Kang Hospital, Kaohsiung, Taiwan; 4 School of Pharmacy, College of Pharmacy, Kaohsiung Medical University, Kaohsiung, Taiwan; 5 Division of General Internal Medicine, Department of Internal Medicine, Kaohsiung Medical University Hospital, Kaohsiung Medical University, Kaohsiung, Taiwan; 6 Department of Laboratory Medicine, Kaohsiung Medical University Hospital, Kaohsiung Medical University, Kaohsiung, Taiwan; 7 Department of Pediatrics, Kaohsiung Medical University Hospital, Kaohsiung Medical University, Kaohsiung, Taiwan; 8 Department of Laboratory Medicine, School of Medicine, College of Medicine, Kaohsiung Medical University, Kaohsiung, Taiwan

## Abstract

The risk of active tuberculosis (TB) in patients with dermatomyositis (DM) is poorly understood. The cohort study aimed to investigate the association between DM and the risk of active TB disease. We conducted a population based study on 4,958 patients with newly diagnosed DM and 19,832 matched controls according to age, sex, and index date between 1998 and 2008. The hazard ratios (HRs) and cumulative incidences of active TB disease between DM patients and controls were analyzed. During the study period, a total of 85 (1.7%) DM patients developed active TB disease, which was significantly higher than that of non-DM patients (0.64%). The incidence rate of active TB disease was higher among DM patients than controls (incidence rate ratio 2.95; 95% confidence interval [CI], 2.24 to 3.88). The Cox regression model demonstrated significantly higher active TB disease rate among DM patients compared with controls (adjusted HR, 2.64; 95% CI, 1.97 to 3.54; p < 0.001) after adjusting for age, sex, and underlying medical disorders. The most significant risk factors for developing active TB included male sex, diabetes mellitus comorbidity, and use of corticosteroids and azathioprine in DM patients. In conclusion, DM patients are at a greater risk for active TB disease.

Dermatomyositis (DM) is systemic autoimmune rheumatic disease characterized by chronic inflammation of the skeletal muscles, which is frequently accompanied by characteristic skin rashes. In DM patients, ten-year survival rates are reported to be widely variable, ranging from 53–91%^1^,^2^,^3^, and infection is considered one of the leading causes of mortality^1^,^2^,^4^. Several factors, such as immunosuppressive medication use and DM-induced immune system dysfunction, may increase infection susceptibility in DM patients^1^,^4^,^5^.

Active tuberculosis (TB) infection is a major health problem and remains the leading cause of death by infectious disease worldwide^6^,^7^. Active TB can develop either from primary progressive tuberculosis or reactivation of latent tuberculosis infection (LTBI). In Taiwan, the incidence of TB among the general population is 74.6 cases per 100,000 population-year^8^. Patients with systemic autoimmune diseases, including systemic lupus erythematosus^9^,^10^,^11^, rheumatoid arthritis^12^,^13^, systemic sclerosis^14^, and Sjogren’s syndrome^15^, are at a higher risk of acquiring active TB. However, only a few case series reports regarding the risk of active TB disease in DM patients have been published^16^,^17^. The association between the risk of active TB disease in patients with DM and the possible causative risk factors, comorbidities and medication effects remains unclear. To evaluate the risk factors that are associated with the development of active TB disease in DM patients, a database acquired from the Taiwan National Health Research Institute (NHRI) was used to investigate the incidence and treatment modalities of DM patients who developed TB.

## Methods

### Data Source

This study was based on a longitudinal health insurance database, the National Health Insurance Research Database (NHIRD), provided by the Taiwan National Health Research Institute. In 1995, Taiwan launched a compulsory social insurance program, the National Health Insurance (NHI) program, to provide health care for the entire population of the island. The annual coverage rate of the NHI program ranges from 96.16% to 99.6% and includes contracts with 97% of all hospitals and clinics; more than 23 million Taiwanese residents have enrolled since 1997. The program covers all medical benefit claims of ambulatory and inpatient care and has been extensively used for many epidemiological studies. The NHIRD established a registry system for “catastrophic illnesses”, including cancer, end-stage renal disease, congenital illness, and several autoimmune diseases. Insured persons with major diseases can apply for catastrophic illness registration cards from the Bureau of National Health Insurance (BNHI) and do not need to make co-payments when seeking health care for their illness. Both the outpatient and inpatient claims of beneficiaries with a catastrophic illness certificate are collected in a catastrophic illness profile and are distributed as a package. The BNHI performs routine validation of the diagnoses by reviewing the original medical charts of all patients who apply for catastrophic illness registration. In this study, all cases of DM patients were obtained from the Registry of Catastrophic Illness Database. The database includes all relevant information from the status of the catastrophic illness certificate, such as diagnostic codes in the format of the International Classification of Disease, Ninth Revision, Clinical Modification (ICD-9-CM), date of diagnosis, date of death, date of every clinic visit, details of prescriptions, expenditure amounts, and outpatient and inpatient claim data for the beneficiaries with catastrophic illnesses. This study was approved by the research ethics board of Kaohsiung Medical University Hospital (KMUHIRB-EXEMPT(I)-20150037).

### Diagnostic criteria of DM

For a DM-related catastrophic illness to be certified, a DM patient is required to have had symptoms of symmetrical proximal muscle weakness and associated dermatological manifestations (including a heliotrope rash, Gottron sign, and Gottron papules). Additionally, electromyography results, muscular biopsy results, levels of muscle enzymes (creatine kinase), and levels of lactate dehydrogenase, aspartate aminotransferase and alanine aminotransferase are reviewed. The DM diagnosis and coding in the Catastrophic Illness Database are accurate.

### Definition of TB and comorbidities

A principal diagnosis of TB was defined by using compatible ICD-9-CM codes of TB (010–018) to assess patients who underwent at least two ambulatory visits or hospitalizations. The diagnosis was further confirmed by medical records that included simultaneous prescriptions for two or more types of anti-TB drugs for >60 days over a 180-day period^18^. The anti-TB drugs that were used included isoniazid, ethambutol, rifampin, pyrazinamide, prothionamide, cycloserine, aminogylcoside (streptomycin, amikacin, and kanamycin), and quinolone (levofloxacin and moxifloxacin). In Taiwan, TB disease is an infectious disease of obligatory notification. When a patient is diagnosed with incident TB and is prescribed anti-TB drugs, the diagnosing physician is required to declare the case to the Taiwan Center for Disease Control (CDC) within 1 week. Because it takes 2 months to obtain a TB culture report in clinical practice, in this study, a TB-positive patient was defined as one who was diagnosed with TB and who had insurance claims for anti-TB drugs for more than 60 days.

The comorbidities that were identified in this study included diabetes mellitus (ICD-9-CM code 250), hypertension (ICD-9-CM codes 401–405), chronic kidney disease (ICD-9-CM code 585), chronic obstructive pulmonary disease (COPD) (ICD-9-CM codes 491, 492, and 496), cancer (ICD-9-CM codes 140–208), and alcoholism (ICD-9-CM codes 291, 303, 305.0, 357.5, 425.5, 571.0, 571.1, 571.2, 571.3, 980.0, and V11.3). All of the included comorbidities were defined by disease codes that were based on at least two ambulatory visits or hospitalizations before a principal diagnosis was made (Supplemental Table S1).

## Study Cohorts

A diagnosis of DM (ICD-9-CM code 710.3) was defined according to ACR diagnostic criteria and was confirmed through the Registry for Catastrophic Illness Patient Database. Patients who were newly identified as having DM over the course of January 1, 1998 to December 31, 2007 were included in the DM cohort. The date on which a DM patient registered for catastrophic illness certification was defined as the index date. Individuals younger than 18-years old (n = 276) and those who were diagnosed with TB before the index date (n = 48) were excluded from study. We exclude individuals <18 years because DM in childrens/young adults is different from the adult form of the disorder in several clinical presentations^19^. A comparison cohort was randomly selected from the Longitudinal Health Insurance Database (LHID), which contains 1,000,000 randomly selected NHI beneficiaries. Each DM patient was matched with four non-DM controls according to age, sex, and included index date^20^. In both cohorts, patients who were diagnosed with active TB disease before the index date or whose records were missing age and sex data were excluded from analysis. A total of 4,958 DM patients and 19,832 non-DM controls were enrolled in the study cohort. The follow-up period lasted until diagnosis of active TB disease, death, or the end of 2008.

### Sensitivity analysis

Several sensitivity analyses were performed to assess the robustness of the main findings. The main analysis contained the whole DM cases and 1:4 match controls. Sensitivity analyses included 1) The main analysis enrolled DM patients and controls excluding history of diabetes mellitus; 2) The main analysis enrolled DM patients and controls excluding history of diabetes mellitus, alcoholism; and 3) The main analysis enrolled DM patients and controls excluding history of diabetes mellitus, chronic kidney disease, COPD, cancer and alcoholism. These sensitivity analyses were conducted for the purpose of examining whether the main findings of the study were robust to different assumptions.

## Statistical Analysis

Normally distributed continuous data were expressed as means ± standard deviations. Numerical data with non-normal distributions were expressed as medians and interquartile ranges. All data were expressed as frequency (percentage) or as means ± standard deviations. Student’s t-test was used to compare parametric continuous data between groups, whereas the chi-square test was used for categorical data. The differences between the proportions of individuals who developed TB in the DM and control groups during the 10-year follow-up period were analyzed using the Kaplan-Meier method with a log-rank test. After confirming the assumption of proportional hazards by Schoenfeld residuals trend tests, which examined the interactions between predictors and event time, the Cox proportional hazard model was applied to examine the association of DM with active TB disease. Additionally, multivariate Cox proportional hazard regression was performed to analyze independent risk factors for active TB disease. Hazard ratios (HRs) and 95% confidence intervals (CIs) were determined. Statistical significance was inferred at a two-sided *p* < 0.05. Analyses were performed using the SAS statistical package (version 9·2; SAS Institute Inc., www.sas.com).

## Results

### Demographic data of the study subjects

**Figure 1:**
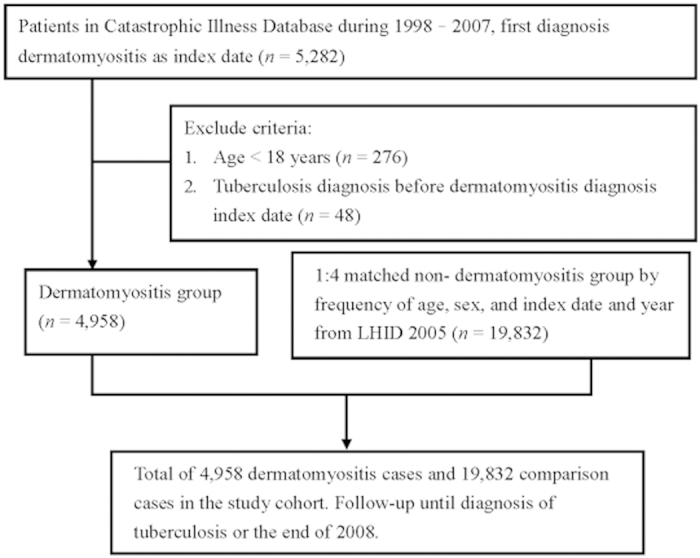
Flow chart of participant recruitment in the National Health Insurance Database, Taiwan, 1998–2007. Footnote: Non- dermatomyositis patients were enrolled from Longitudinal Health Insurance Database (LHID) 2005, which excluded all dermatomyositis diagnostic ICD-9 code (ICD-9CM 710.3). LHID was a cohort, which the National Health Research Institute of Taiwan randomly sampled a representative database of 1,000,000 subjects from the entire National Health Insurance enrollees. There were no statistically significant differences in age, gender, and health-care costs between the sample group and all enrollees.

The study entry flow chart is shown in Fig. 1. A total of 4,958 DM patients were included after excluding individuals who were younger than 18-years old or who had TB prior to DM diagnosis. Patient characteristics and selected comorbid medical disorders at baseline are presented in Table 1. The mean age ± standard deviation (SD) of the study population was 52.89 ± 13.66 years, and 59.9% of the population was comprised of women. DM patients had more comorbidities than controls, including diabetes mellitus, hypertension, chronic kidney disease, COPD, cancer, and alcoholism.

### The incidence rates of TB among DM patients and controls

**Figure 2:**
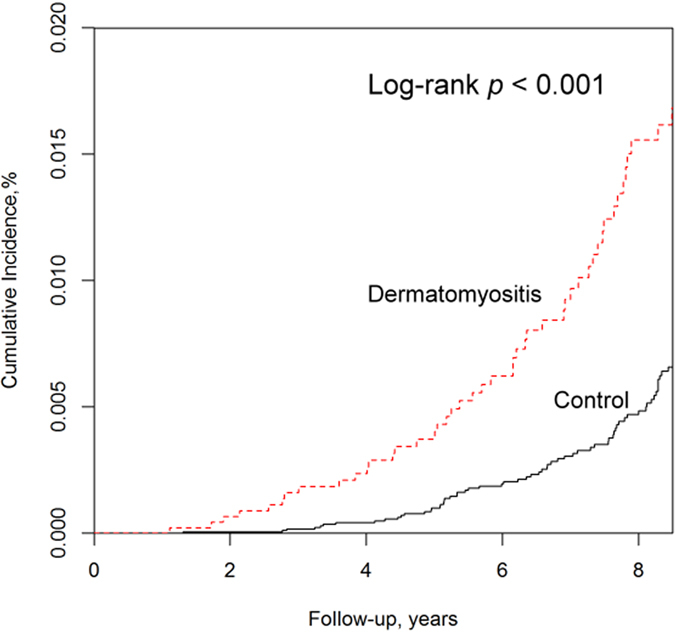
Cumulative incidences of active tuberculosis disease estimated by the Kaplan–Meier approach in patients with and without dermatomyositis. Dermatomyositis groups increased cumulative incidences of active tuberculosis disease than comparison groups (Log rank P < 0.001).

During the follow-up period, TB disease was diagnosed in 85 patients with DM (1.71%), corresponding to an incidence rate of 305 (95% CI = 243–377) per 100,000 person-years. In the non-DM groups, 126 patients had TB disease (0.64%), corresponding to an incidence rate of 103 (95% CI = 86–123) per 100,000 person-years (Table 2). The incidence rates of active pulmonary and extra-pulmonary TB disease were both increased in DM patients versus non-DM patients. The active TB disease risk was significantly higher in DM patients than in non-DM patients (*p* < 0.001), with an incidence rate ratio of 2.95 (95% CI = 2.24–3.88) (Table 2). The cumulative incidences of active TB disease were higher in DM patients than in their matched controls (*p* < 0.001), as determined by the Kaplan-Meier approach (Fig. 2).

### The risk factors associated with active TB disease

As shown in Table 3, we performed multivariable Cox proportional hazards model analysis and found that DM itself was an independent risk factor for TB infection. The adjusted HR for TB disease in DM patients was 2.64 (95% CI, 1.97–3.54; *p* < 0.001) after adjusting for age, sex, location, socioeconomic status, diabetes mellitus, hypertension, chronic kidney disease, COPD, cancer, and alcoholism. Multivariate analysis demonstrated that diabetes mellitus was the leading risk factor for the development of active TB disease (adjusted HR, 2.12; 95% CI, 1.46 to 3.07; *p* < 0.001), followed by male gender (adjusted HR, 1.81; 95% CI, 1.37 to 2.41; *p* < 0.001), and age (adjusted HR, 1.03; 95% CI, 1.02 to 1.04; *p* < 0.001) (Table 3).

### Risk factors for active TB disease in patients with DM

Of 838 patients with DM, 521 (62.2%) received corticosteroids, 102 (12.2%) received cyclophosphamide (CYC), 10 (1.2%) received cyclosporine, 32 (3.8%) received methotrexate, and 46 (5.5%) received azathioprine. Univariate Cox regression analysis identified 4 risk factors for the development of TB in patients with DM: male sex, diabetes mellitus, use of corticosteroids, and use of azathioprine (Table 4). The multivariate Cox proportional hazards analysis indicated that all four of these factors were independent risk factors for TB in DM patients: male sex (adjusted HR 1.81, 95% CI 1.16–2.82; *p* = 0.009), diabetes mellitus (adjusted HR 2.13, 95% CI 1.22–3.72; *p* = 0.007), corticosteroid usage (adjusted HR 1.96, 95% CI 1.01 –3.81; *p* = 0.048), and azathioprine usage (adjusted HR 2.27, 95% CI 1.32–3.89; *p* = 0.003). The use of cyclophosphamide, cyclosporine, and methotrexate did not significantly increase TB risk in DM patients (Table 4).

### Sensitivity analysis

The robustness of this study was tested with a series of sensitivity analyses. The results regarding the impact of DM were consistent among the various subgroups. In all of these analyses, which were based on different assumptions, DM patients were associated with a statistically significantly increased risk of active TB disease when compared to non-DM patients (Supplemental Table S2). Using multivariable Cox proportional hazards model, the adjusted HR for active TB disease in DM patients was 2.89 (95% CI, 2.08–4.00; *p* < 0.001) after excluding history of diabetes mellitus, 2.94 (95% CI, 2.12–4.08; *p* < 0.001) after excluding history of diabetes mellitus and alcoholism, and 3.53 (95% CI, 2.48–5.03; *p* < 0.001) after excluding history of diabetes mellitus, chronic kidney disease, COPD, cancer, and alcoholism.

## Discussion

The impacts of DM on the risk of active TB disease and the details surrounding this association have not been well established. To the best of our knowledge, this is the first study to investigate these matters by using an adjustment for comorbidities in a large-scale cohort. Active TB disease risk in DM patients was found to be significantly higher than in non-DM patients. In addition to DM, the current study also demonstrated that male sex, diabetes mellitus comorbidity, and corticosteroid or azathioprine drug use increased the risk of active TB disease in DM patients.

Our study findings were convincing and may aid in the objective analysis of TB in patients with DM due to our large sample size, strict diagnostic criteria and unbiased subject selection. The diagnosis of TB is based on clinical suspicion followed by chest X-ray and confirmation by smear and culture. In Taiwan, after a definite diagnosis is made, a combined treatment is used for active TB, in accordance with current international guidelines. Therefore, the diagnosis of TB in our study were highly reliable. The stringent definitions of TB disease and DM that were used in this study added strength to the associations that were found between DM and TB disease risk. While historically the development of chronic respiratory symptoms in DM patients has always led to the consideration of interstitial lung disease, the results of our study should urge primary health care providers to carefully monitor DM patients who present with chronic cough, fever, or other manifestations of TB disease.

In our study, patients with DM were at a greater risk for developing active TB compared to controls. Ramagopalan *et al.* reported that patients with DM had significantly elevated risks of acquiring TB following hospital admission^21^. Previous reports have demonstrated an association between several immune-mediated diseases and a subsequent risk of acquiring TB^22^,^23^,^24^. In these studies, possible mechanisms that may have led to increased TB risk includes the immune dysfunction that results from immune-mediated diseases and the effects of the immunosuppressant drugs that are used to treat them^21^. We also found that diabetes mellitus was an independent risk factor for developing active TB. This finding is consistent with previous studies^25^,^26^.

We observed that patients who had received corticosteroid and azathioprine prescriptions were at a higher risk of developing active TB disease. Corticosteroids have long been associated with the risk of acquiring TB^27^,^28^,^29^. This may be because corticosteroids have profound effects on immunomodulation, including inhibition of macrophage function^30^, the induction of apoptosis in dendritic cells^31^ and the suppression of T cell activation^32^. These complex effects may attenuate host immune responses and predispose patients to TB infection^27^. For more than 30 years, azathioprine, an immunosuppressive drug that is a derivative of 6-mercaptopurine agent, has been used both as a monotherapy and also as an adjuvant therapy to prednisolone to treat patients with DM^33^. In the present study, DM patients using azathioprine exhibited a 2.27-fold increased risk of developing active TB after adjusting for covariates. It has been also demonstrated that azathioprine acts at several different levels to affect lymphocyte-mediated cellular immunity, and its possible mechanisms include a reduction in macrophage activation^34^ and inhibition of lymphocyte activation, stimulation and mixed lymphocyte reactions^35^,^36^,^37^,^38^. Gorski *et al.* discovered that higher azathioprine concentrations impair T-helper function and B-cell differentiation in peripheral blood, lymph nodes, and spleen lymphocytes^39^. Selective effects of azathioprine on T-cell subpopulations that regulate B-cell antibody responses have also been demonstrated^40^. These reports provide a possible mechanism to explain how the use of azathioprine may increase the risk of developing active TB.

This study had several limitations. First, it was a retrospective cohort study based on diagnostic codes and prescription history. Therefore, the possibility of an ascertainment bias cannot be excluded in observational study design. Patients with DM may have greater medical attention toward other health problems and increase exposure to the medical community which might have more chance of discovering other diseases. This bias could lead to an increase in the diagnosed rate for active TB among DM patients compared with the unaffected controls. However, because of the mandatory medical insurance and high accessibility of medical services in Taiwan, the chance of active TB disease being overlooked should be minimal. Furthermore, the standard procedure for diagnosing TB disease (acid-fast stain and culturing) was not included. Instead, in this study, the diagnosis of TB disease was determined based on whether a patient had taken anti-TB medication for more than 60 days. It is likely that some patients with TB disease did not submit claims for medication or died before 2 months of treatment had elapsed. Nonetheless, the incidence of TB disease in DM patients was higher than in the general population of Taiwan^8^,^41^,^42^. However, it is difficult to distinguish cases of new infections from those associated with the reactivation of old TB lesions. Second, the data notably lacked information regarding individual severity levels of DM. People with DM who had minor disease or who had never sought medical attention would not have received a critical illness certificate and as such these patients were not included in our study. Therefore, the incidence of DM that was reported in this study may have been underestimated. Finally, as it is an administrative dataset, our database lacked several data points with regard to the individuals who were included, including tobacco use, alcohol consumption, nutritional status, intravenous drug abuse, occupational exposure, and history of contact with TB; each of these factors may contribute to TB disease. These factors may lead to potential residual confounding, which could have affected the interpretation of our findings. To prevent potential bias in our study, related major and common medical comorbidities were adjusted.

## Conclusion

In summary, our findings provide solid epidemiological evidence that DM patients are at a greater risk for active TB disease, especially male patients and patients with diabetes mellitus comorbidity, as well as those who have been prescribed corticosteroids and azathioprine. Rheumatologists and primary health care providers should be aware that DM patients have a significant risk of developing active TB.

## Additional Information

**How to cite this article**: Wu, P.-H. *et al.* The increased risk of active tuberculosis disease in patients with dermatomyositis - a nationwide retrospective cohort study. *Sci. Rep.* **5**, 16303; doi: 10.1038/srep16303 (2015).

## Supplementary Material

Supplementary Information

Supplementary Tables

## Figures and Tables

**Table 1:** Clinical characteristics between dermatomyositis groups and age-, sex- matched comparison groups in Taiwan between 1998 and 2007.

	Dermatomyositis Groups(n = 4958)		Comparison Groups(n = 19,832)		P value
	n	%	N	%	
Age, years					1
18–39	1046	21.1	4184	21.1	
40–49	1081	21.8	4324	21.8	
50–59	989	19.9	3956	19.9	
≧60	1842	37.2	7368	37.2	
Gender					1
Male	1989	40.1	7956	40.1	
Female	2969	59.9	11876	59.9	
Area					0.058
City	3735	75.3	14,679	74	
Rural area	1223	24.7	5153	26	
Socioeconomic status					<0.001
Dependent	1504	30.3	5753	29	
NT$ <20000	1362	27.5	4470	22.5	
NT$ ≧ 20000	2092	42.2	9609	48.5	
Comorbidity					
Diabetes mellitus	711	14.3	1731	8.7	<0.001
Hypertension	1358	27.4	3666	18.5	<0.001
Chronic kidney disease	195	3.9	139	0.7	<0.001
COPD	722	14.6	1341	6.8	<0.001
Cancer	735	14.8	475	2.4	<0.001
Alcoholism	84	1.7	79	0.4	<0.001

COPD, Chronic obstructive pulmonary disease.

**Table 2:** Incident rate of active tuberculosis disease among dermatomyositis and comparison groups.

Clinical Outcome	dermatomyositis groups (*n* = 4958)			comparison groups (*n* = 19,832)			Incidence Rate Ratio	95% CI
	No.	IncidenceRate^a^	95% CI	No.	IncidenceRate^a^	95% CI		
All active tuberculosis	85	305	243–377	126	103	86–123	2.95	2.24–3.88
Active pulmonary TB incident rate	43	154	111–208	115	94.3	78–113	1.64	1.15–2.32
Active extrapulmonary TB incident rate	42	151	109–204	11	555	277–993	2.72	1.40–5.27

Abbreviation: HCV, hepatitis C; IR, incident rate; TB, tuberculosis.

^a^Incidence of TB: Per 100,000 person-years.

**Table 3:** The risk factors associated with active tuberculosis disease among all the enrollees by univariate and multivariate analysis cox regression analysis.

Variables	Multivariate analysis^a^		
	HR	95% CI	*p*Value
Dermatomyositis	2.64	1.97–3.54	<0.001
Age (per year)	1.03	1.02–1.04	<0.001
Male sex	1.81	1.37–2.41	<0.001
City Area	0.77	0.57–1.04	0.089
Socioeconomic status			
Low economics	1.21	0.87–1.70	0.256
Moderate economics	1.09	0.76–1.56	0.635
Diabetes mellitus	2.12	1.46–3.07	<0.001
Hypertension	1.25	0.87–1.79	0.222
Chronic kidney disease	1.20	0.46–3.13	0.704
COPD	1.16	0.75–1.78	0.506
Cancer	1.45	0.87–2.40	0.506
Alcoholism	1.31	0.39–4.44	0.660

Abbreviation: CI, confidence interval; COPD, Chronic obstructive pulmonary disease; HR, hazard ratio; TB, tuberculosis.

^a^Each variable was adjusted for every other variable listed.

**Table 4:** Cox regression of risk factors for tuberculosis in the dermatomyositis patients.

Variables	Multivariate analysis^a^		
	HR	95% CI	*p*Value
Age ≧ 60 years	1.39	0.84–2.30	0.199
Male sex	1.81	1.16–2.82	0.009
Comorbidities			
Diabetes mellitus	2.13	1.22–3.72	0.007
Hypertension	1.06	0.60–1.88	0.835
Chronic kidney disease	1.37	0.40–4.72	0.537
COPD	0.89	0.46–1.72	0.770
Cancer	1.52	0.81–2.83	0.197
Alcoholism	0.63	0.08–5.08	0.663
Medications, ≧ 28cDDDs			
Corticosteroid	1.96	1.01–3.81	0.048
Cyclophosphamide	1.27	0.67–2.41	0.468
Cyclosporine	1.04	0.43–2.51	0.935
Methotrexate	1.07	0.60–1.91	0.829
Azathioprine	2.27	1.32–3.89	0.003

Abbreviation: CI, confidence interval; COPD, Chronic obstructive pulmonary disease; HR, hazard ratio; TB, tuberculosis.

^a^Each variable was adjusted for every other variable listed.
