# Supplementary material for: The increased risk of active tuberculosis disease in patients with dermatomyositis – a nationwide retrospective cohort study
Source: Sci Rep. 2015 Nov 17;5:16303. doi: 10.1038/srep16303 (PMC4647179; doi:10.1038/srep16303)
Supplement: Supplementary Tables [file srep16303-s2.pdf]

Manuscript Title: The increased risk of active tuberculosis disease in patients with dermatomyositis - a nationwide retrospective cohort study

Authors, Institution and Affiliations:

Ping-Hsun Wu[1, 2], Yi-Ting Lin[3], Yi-Hsin Yang[4], Yu-Chih Lin[5], Yi-Ching Lin[6, 7, 8]

[1]Division of Nephrology, Department of Internal Medicine, Kaohsiung Medical University Hospital, Kaohsiung, Taiwan

[2]Faculty of Internal Medicine, College of Medicine, Kaohsiung Medical University, Kaohsiung, Taiwan

[3]Department of Family Medicine, Kaohsiung Municipal Hsiao–Kang Hospital, Kaohsiung, Taiwan

[4]School of Pharmacy, College of Pharmacy, Kaohsiung Medical University, Kaohsiung, Taiwan

[5]Division of General Internal Medicine, Department of Internal Medicine, Kaohsiung Medical University Hospital, Kaohsiung Medical University, Kaohsiung, Taiwan

[6]Department of Laboratory Medicine, Kaohsiung Medical University Hospital, Kaohsiung Medical University, Kaohsiung, Taiwan

[7]Department of Pediatrics, Kaohsiung Medical University Hospital, Kaohsiung Medical University, Kaohsiung, Taiwan

[8]Department of Laboratory Medicine, School of Medicine, College of Medicine, Kaohsiung Medical University, Kaohsiung, Taiwan

Corresponding author:

Yi-Ching Lin, MD

Department of Laboratory Medicine, Kaohsiung Medical University Hospital, Kaohsiung Medical University, No. 100, Zihyou 1st Road, Sanmin District, Kaohsiung 807, Taiwan.

Email: winterjeanne@gmail.com

TEL: +886-7-3121101 ext.7230
FAX: +886-7-3121101 ext.7267

Supplemental Table S1. The corresponding ICD-9 codes for the diagnoses of diseases examined in this study

Diagnosis	Corresponding ICD-9 codes
Dermatomyositis	【710.3】 plus certificate of catastrophic illness
Tuberculosis	【010】 ~ 【018】 plus 2 or more types of anti-tuberculosis drugs for more than 2 months
Pulmonary tuberculosis	【010】 ~ 【012】
Extra-pulmonary tuberculosis	【013】 ~ 【018】
Diabetes mellitus	【250】
Hypertension	【401】 ~ 【405】
Chronic kidney disease	【585】
COPD	【491】【492】【496】
Cancer	【140】 ~ 【208】
Alcoholism	【291】【303】【305.0】【357.5】【425.5】【571.0】【571.1】【571.2】【571.3】【980.0】【V11.3】

Abbreviations: ICD, international classification of disease; COPD, chronic obstructive pulmonary disease

Supplemental Table S2. Sensitivity analyses

	Dermatomyositis Groups		Comparison groups				
	No. of active TB disease	Total no. of patients	No. of active TB disease	Total no. of patients	Adjusted HR	95% CI	*p* Value
Main analysis	85	4958	126	19832	2.64	1.97 - 3.54	<0.001
Main analysis excluding history of diabetes mellitus	66	4247	104	18101	2.89	2.08 - 4.00	<0.001
Main analysis excluding history of diabetes mellitus, alcoholism	65	4184	103	18037	2.94	2.12 - 4.08	<0.001
Main analysis excluding history of diabetes mellitus, chronic kidney disease, COPD, cancer, alcoholism	49	3030	87	16578	3.53	2.48 - 5.03	<0.001

Abbreviation: HR, hazard ratio; CI, confidence interval; COPD, Chronic obstructive pulmonary disease
